# Association of Depression, Poor Mental Health Status and Asthma Control Patterns in US Adults Using a Data-Reductive Latent Class Method

**DOI:** 10.7759/cureus.33966

**Published:** 2023-01-19

**Authors:** Chukwuemeka E Ogbu, Jayashree Ravilla, Menkeoma Laura Okoli, Onyekachi Ahaiwe, Stella C Ogbu, Eun Sook Kim, Russell S Kirby

**Affiliations:** 1 College of Public Health, University of South Florida, Tampa, USA; 2 Internal Medicine, Monmouth Medical Center, Long Branch, USA; 3 Epidemiology and Public Health, The University of Texas Health Science Center at Houston, Houston, USA; 4 Biomedical Sciences, Tulane University School of Medicine, New Orleans, USA; 5 College of Education, University of South Florida, Tampa, USA

**Keywords:** epidemiology and biostatistics, latent class analysis, poor mental health, depression, asthma control

## Abstract

Objectives

To explore the association between depression, poor mental health status, and asthma control patterns among US adults using a latent class analysis (LCA) approach.

Methods

We used data from 10,337 adults aged 18 years and above from the 2016 Behavioral Risk Factor Surveillance System (BRFSS) Asthma Call-back Survey. Data-reductive LCA was used to derive asthma control patterns in the population using class variables indicative of asthma control. Besides univariate analysis, adjusted and unadjusted logistic regression models were used to examine the association of depression and poor mental health on the derived asthma control patterns.

Results

About 27.8% of adults aged <55 reported depression, while 27.3% aged ≥55 years were depressed. The latent class prevalence of asthma control patterns was 42.8%, 31.1%, and 26.1%, corresponding to good, fair, and poor asthma control patterns, respectively. In adults aged <55 years, odds of depression (OR=1.52, 95% CI=1.27-1.82) and poor mental health (OR=1.58, 95% CI=1.27-1.96) were higher in the poor asthma control group compared to the good asthma control group. Odds for depression (OR=1.28, 95% CI=1.06-1.53) were also higher in the moderate asthma control group compared to the good asthma control group. Among those aged ≥55 years, depression odds (OR=1.57, 95% CI=1.31-1.87) were higher in only the poor asthma control group.

Conclusions

These findings may have public health implications. Detecting, screening, and treating depression and mental health disorders may help improve asthma control in people with asthma.

## Introduction

Asthma is a chronic inflammatory airway disease and the leading cause of years lived with disability in the United States and worldwide, with greater prevalence among adults [1, 2]. Despite the best efforts to mitigate the risk of asthma and asthma exacerbations through effective treatments and control, asthma is still a principal cause of morbidity and mortality in the adult population [2, 3]. The prevalence of psychopathology, specifically depression and mental health impairment, is high among this population [4, 5, 6]. Therefore, adults' asthma, depression, and mental health problems are public health issues. The association between depression and asthma has increasingly been recognized [7, 8]. For instance, a systemic review of epidemiological studies has shown that depression is more common in patients with asthma compared to the general population [8], leading to the notion that asthma may be a psychosomatic disease [9]. Several studies suggest that depression increases the risk of asthma exacerbation [10, 11]. While the causal relationship between asthma and depression is not fully understood, a meta-analysis of prospective studies indicates that depression affects asthma through inflammation, neuroendocrine effects, and shared behavioral factors like smoking and obesity [12]. Depression is also associated with asthma morbidities [13], lower quality of life [14], asthma symptoms [15], and increased asthma healthcare utilization [16]. Therefore, detecting and preventing depression in adults with asthma is important in asthma control. However, few studies have examined this association across adult populations, given that there are differences in the prevalence of depression and other mental health disorders among older and younger adults [6].

More so, the current strategy used to classify asthma patients has been divided into investigator-imposed and data-driven approaches [17]. The former is known as the hypothesis-driven method, while the latter is the hypothesis-generating approach [18]. The hypothesis-driven method accounts for a person's pattern of symptom change, environmental triggers, or airway pathology and then classifies patients into "asthma phenotypes" [18]. Clinically standardized and validated questionnaires that include Asthma Control Questionnaire, Childhood Asthma Control Test, Asthma Control Test, Asthma Therapy Assessment, and Asthma Scoring System are used to assess the hypothesis-driven phenotype and measure asthma severity and control in population and clinical asthma studies [19]. However, this approach may not be able to estimate the uncertainty of asthma phenotypes [18]. While the data-driven approaches have been applied to understanding asthma phenotypes, they have yet to be applied to understanding asthma control patterns in the population. They could represent a new way of assessing asthma control using novel means that are not based on diagnostic testing. Data-driven methods incorporating novel statistical methods may help better understand population asthma control from datasets that do not have clinical measures of asthma disease severity. Therefore, our study aims to examine the association between depression and poor mental health status with asthma control patterns among US adults using a latent class analysis (LCA) approach. The LCA is a data-reductive person-centered approach that involves the creation of discrete latent categories that represent patterns of asthma control in the population, thereby classifying similar persons that consist of homogenous individuals with regard to the observed variables [20].

## Materials and methods

We used the 2016 Behavioral Risk Factor Surveillance System (BRFSS) Asthma Call-back Survey for this analysis. The BRFSS Adult Asthma Call-back Survey (ACBS) is a cross-sectional study of non-institutionalized US adults aged 18 years and above. The BRFSS is a state-based surveillance system that collects information on preventive health practices, health risk behaviors, health care access and utilization related to chronic disease and injuries, and preventable infectious disease and is sponsored by the CDC. The ACBS is conducted two weeks after the BRFSS and is a continuous survey that provides yearly data about people with asthma, including demographics and illness histories. BRFSS adult respondents who reported a history of asthma diagnosis are eligible for the ACBS and are randomly selected to participate. A couple of questions were used to measure asthma prevalence in BRFSS. An affirmative response to the questionnaire items "Have you ever been told by a doctor, nurse, or other health professionals that you have asthma?" and "Do you still have asthma?" were used to denote lifetime asthma and current asthma status, respectively. An affirmative response to the first question was a criterion for participating in the follow-up telephone Asthma Call-back. Response rates for ACBS are calculated using Council of American Survey and Research Organizations guidelines [21]. The median ACBS response rate among the 31 participating states and DC participating in the 2016 ACBS was 44.53% (36.9-60.0%) [21]. Our dataset consisted of 13,922 adults sampled from the 31 states of the United States, DC, and Puerto Rico in 2016. A total of 10,337 adults remained after excluding adults diagnosed with chronic obstructive pulmonary disease, emphysema, and bronchitis due to the overlap of these diseases with asthma [22]. ACBS received approval from the States' Institutional Review Boards and Ethics Review Board of the Asthma and Community Health Branch in the National Center for Environmental Health. All participants provided informed consent [22]. This study is exempt from the full institutional review board process because the ACBS is a public-use dataset. A detailed description of the data, sampling method and other analytical guidelines is available elsewhere [23].

Assessments 

Class variables were selected and re-categorized from the dataset after identifying history-elicited asthma control markers, including symptoms and functional limitation, medication use, and asthma-related medical resource utilization [19, 24, 25]. These variables were the presence of asthma symptoms (cough, wheezing, and dyspnea) in the past 30 days, difficulty sleeping due to asthma in the past 30 days, ever had an asthma episode or attack during the past 12 months, ever visited the ER or urgent care because of an asthma attack, ever treated in the ER by a physician for worsening asthma symptoms/episodes/attacks, ever had at least one asthma episode/attack in the past three months, ever had activity limitation due to asthma in the past 30 days, ever taken a prescription medicine using an inhaler in the last three months, ever taken any medicine in pill form for asthma in the past three months, ever used a nebulizer for asthma in the past three months and active status. Responses were coded as binary "yes" and "no" responses. Depression was assessed with the question, "Has a doctor, nurse, or other health professionals ever told you that you have a depressive disorder, including depression, major depression, dysthymia, or minor depression?". A "yes" response signify depression. A "none to less than 14 days" or "14 or more days" response to the survey item and "Now thinking about your mental health, stress, depression, and problems with emotions, for how many days during the past 30 days was your mental health not good?" was used to ascertain poor mental health days. Other covariates included age (<55 years or ≥55 years), sex (male vs. female), smoking status (current smoker vs. former/never smoker), mold in the house (yes vs. no), race (white non-Hispanic, black, Hispanic, multiracial), and income (<$25000/year or ≥$25,000/ year and above).

Statistical analyses

The adult ACBS uses a stratified, multistage complex survey design to enhance the representativeness of the US adult population. Analytical guidelines using complex analytical procedures suggested by the ACBS were followed, and appropriate weighting was applied to account for the sampling strata, cluster, and primary sampling unit (PSU).

First, we conducted a descriptive analysis of our study population. The Rao-Scott chi-square test was used to conduct a bivariate analysis between independent variables (history elicited asthma control markers) and the dependent variable (depression) and covariates. LCA models were constructed with a number of subclasses ranging from one to six. A null model was first fitted without grouping variables (age) and covariates to understand the overall subclass structure. These models were then checked for model fitness, parsimony, and interpretability of the LCA solutions [20]. The final model was assessed by the number of optimal parameters, Bayesian Information Criterion (BIC), sample size Adjusted Bayesian Information Criteria (ABIC), Akaike Information Criterion (AIC), adjusted Lo-Mendell-Rubin adjusted likelihood ratio test (LMR-LR test), entropy, and interpretability of the latent class solutions [26, 27]. The smaller AIC and BIC and higher entropy values suggest a better model. An LMR-LR test that is statistically significant (p<0.05) indicated a substantial improvement in the model fit of an n model compared with the (n-1) class model. A descriptive analysis of the asthma control markers' item response probabilities (probability of a "yes" response) was also conducted, and probabilities >0.50 was used as a cut for a high or low endorsement of that particular asthma control variable.

Age was used as a grouping variable to stratify selected latent classes to ascertain if latent classes were identical between younger and older adults. We used convenient age categories of <55 years and ≥55 years based on age categories in the dataset. We grouped the latent classes by age, and all parameters were freely estimated. The parameters were also estimated when measurement invariance by age was imposed. The difference was statistically significant, providing evidence that measurement invariance does not hold across age groups for the prevalence of subclasses of asthma control. Therefore, we have separately reported the effect of the exposures on the outcome for these two populations (<55 years and ≥55 years).

The association between asthma control subclasses, depression, and poor mental health status was then evaluated by a multinomial logistic regression with the lower risk class (in this case, adults with "good asthma control pattern") as reference [28]. Odds ratio (OR) and 95% CI were calculated to indicate the increase in odds of membership in a particular latent class relative to the reference. Analyses were conducted using the PROC LCA add-on in SAS version 9.4 and Mplus version 7.

## Results

Males accounted for 43.6% of adults under 55 and 34% of adults above 55 years. White non-Hispanics accounted for 60% of people aged <55 and 73% of those aged ≥55. About 27.8% of adults <55 and 27.2% of adults ≥55 had depression. A total of 81% of those under 55 endorsed poor mental health, while 88% of those ≥55 had <14 days of poor mental health. We showed proportions of adults aged <55 and ≥55 years with each marker of asthma control in Table 1. The results indicate that, except for having visited the ER in the past year because of worsening asthma symptoms, the prevalence of all other selected asthma control markers was more common in adults ≥55 years than in adults <55.

**Table 1:** Characteristics of the study population stratified by age group. * Weighted percentages.

Predictors (*%)	Age < 55 years (n=4707) (%*)	Age ≥55 (n= 5630) (%*)	P-value
Males (%)	1956 (43.64)	1729 (34.64)	<0.0001
Non-Hispanic Whites (%)	3398 (60.47)	4606 (72.90)	<0.0001
Current smokers- Yes (%)	790 (15.18)	354 (7.34)	<0.0001
Binge Drinking -Yes (%)	893 (22.04)	340 (8.48)	<0.0001
Physical health – Good (%)	4018 (87.11)	4497 (79.55)	<0.0001
Poor mental health - Yes (%)	3799 (81.59)	4970 (88.62)	<0.0001
Had a diagnosis of major depression – Yes (%)	1471 (27.82)	1498 (27.23)	0.75
Mold in home- Yes (%)	477 (9.79)	439 (7.91)	0.14
Indoor smoking exposure – Yes (%)	529 (13.10)	362 (7.17)	<0.0001
Income less than 25k – Yes (%)	1151 (29.16)	1410 (27.91)	0.53
Asthma control markers			
Had at least one asthma symptom during the past 30 days (%)	1994 (40.35)	2513 (46.73)	0.0018
Had difficulty sleeping at night due to asthma in the past 30 days (%)	814 (16.26)	905 (18.64)	0.14
Had an asthma episode/attack in the past 12 months (%)	1564 (30.56)	1751 (32.11)	0.43
Visited the ER because of an asthma attack (%)	636 (12.76)	793 (14.30)	0.26
Visited the ER due to worsening symptoms (%)	395 (8.13)	345 (6.56)	0.17
Had an asthma episode attack in the past three months (%)	1173 (23.56)	1232 (23.26)	0.08
Had activity limitation due to asthma in the past 30 days (%)	1337 (27.54)	1728 (32.23)	0.015
Took an inhaler medicine in the past three months (%)	1856 (35.74)	2582 (46.46)	<0.0001
Took asthma med in pill form in the past three months (%)	412 (6.60)	746 (13.63)	<0.0001
Used a nebulizer for asthma in the past three months (%)	337 (5.38)	428 (6.52)	0.16
Asthma status is active (%)	3230 (65.26)	4206 (76.68)	<0.0001

Number of latent clusters

We considered a 3-class model optimal because it had the highest entropy and interpretation compared to the others (Table 2).

**Table 2:** Table of model selection and diagnostics. AIC: Akaike information criterion; BIC: Bayesian information criterion; SAIC: Simultaneous equations model; LMR-LT: Lo-Mendell-Rubin adjusted likelihood ratio test.

# of LC	Loglikelihood	Best H0 Replicated	# parameters	AIC	BIC	SAIC	LMR-LRT (p)	Entropy
1	-55125.01	Yes	11	110272.02	110351.70	110316.74	N/A	N/A
2	-42186.33	Yes	23	84418.65	84585.25	84512.16	25646.16 (p<0.0001)	0.899
3	-39131.97	Yes	35	78333.95	78587.47	78476.24	6054.13 (p <0.0001)	0.923
4	-38175.61	Yes	47	76445.23	76785.67	76636.31	1895.63 (p< 0.0009)	0.912
5	-37735.20	Yes	59	75588.39	76015.76	75828.26	872.97 (p<0.67)	0.862
6	-37404.52	Yes	71	74951.04	75465.32	75239.69	655.45(p <0.22)	0.875

Table 3 shows asthma control markers' item response probabilities (probability of a "yes" response). These probabilities were the basis for interpretation and assigning the latent classes. The greater probabilities appear in bold font to highlight the overall pattern. Latent class 1 has an estimated prevalence of 42.8%. This group of adults endorsed the lowest probabilities in all asthma control markers. They have been designated as adults with "good asthma control" and are, therefore, the lowest risk group. The second class, designated as "fair asthma control," with a prevalence of 31.1%, was the second largest class. Participants in this class endorsed having had asthma in the last 30 days, using inhaler medicine in the past three months, and belonging to the category of those with active asthma status. The third latent was designated the "poor asthma control" group with a prevalence of 26.1%. This group endorsed a higher probability of most of the asthma history elicited markers that include having had at least one asthma symptom, difficulty sleeping at night, having had an asthma attack, and visiting the ER at least once in the past year for both asthma and non-asthma related reasons, use of inhaler medications, and active asthma status.

**Table 3:** Latent class prevalence and item response probability of asthma control markers for each subclass. The greater probabilities appear in bold font to highlight the overall pattern.

	Good asthma control	Fair asthma control	Poor asthma control
Prevalence of latent class	42.8%	31.1%	26.1%
Item-response probabilities of asthma control markers			
Had at least one asthma symptom during past 30 days	0.0001	0.6717	0.8198
Had difficulty sleeping at night due to asthma in past 30 days	0.0001	0.2218	0.3907
Had an asthma episode/attack in the past 12 months	0.0262	0.1192	0.999
Visited the ER at least once in the past one year due to asthma	0.000	0.000	0.919
Visited the ER at least once in the past one year for worsening asthma attack	0.0154	0.3885	0.6198
Had an asthma episode attack in the past three months	0.0004	0.1202	0.3647
Had activity limitation due to asthma in the past 30 days	0.000	0.0556	0.2277
Took an inhaler medicine in the past three months	0.000	0.5859	0.7903
Took asthma med in pill form in the past three months	0.000	0.1169	0.1963
Used a nebulizer for asthma in the past three months	0.000	0.0525	0.1595
Thinks asthma status is active	0.2654	0.9983	0.9995

Association of depression and poor mental health status with latent class membership

Multinomial logistic regression was used to examine the association of depression and poor mental health on asthma control patterns (latent class membership) using the low-risk class as the reference. In the adjusted analysis, adults <55 in the poor asthma control group were more likely to be depressed (OR=1.52, 95% CI=1.27-1.82) and have poor mental health (OR=1.58, 95% CI=1.27-1.96) than those in the low-risk group. For adults <55 years, those in the fair asthma control group were more likely to be depressed (OR=1.28, 95% CI=1.06-1.53) compared to the low-risk group. However, the association with poor mental health was not statistically significant (OR=1.15, 95% CI=0.91-1.45). Being depressed was associated with significantly higher odds of belonging to the latent class 2 (poor asthma control group) compared to latent class 1 (good asthma control) among adults ≥55 (OR=1.57, 95% CI=1.31-1.8). Compared to the well-controlled asthma group, depression and poor mental health were not statistically significant among adults ≥55 in the fair asthma control group (Table 4). 

**Table 4:** Unadjusted and adjusted multinomial logistic regression of depression and poor mental health on latent class membership, BRFSS Asthma Call-back Survey, 2016. a. Adjusted model controlled for sex, race, income, mold in the home, indoor smoke exposure, smoking status, binge drinking, and physical health status. BRFSS: Behavioral Risk Factor Surveillance System.

Covariates	Good asthma control (LC 1)	Poor asthma control (LC 2)	Fair asthma control (LC 3)
Unadjusted OR and 95% CI			
Age <55			
Depression	Ref	2.12 (1.82-2.48)	1.55 (1.32-1.82)
Poor mental health	Ref	2.25 (1.87-2.70)	1.38 (1.13-1.68)
Age ≥ 55			
Depression	Ref	1.99 (1.70-2.33)	1.16 (1.00-1.36)
Poor mental health	Ref	1.89 (1.50-2.37)	1.20 (0.95-1.52)
Adjusted OR and 95% CI			
Age <55			
Depression ^a^	Ref	1.52 (1.27-1.82)	1.28 (1.06-1.53)
Poor mental health ^a^	Ref	1.58 (1.27-1.96)	1.15 (0.91-1.45)
Age ≥ 55			
Depression ^a^	Ref	1.57 (1.31-1.87)	1.06 (0.89-1.26)
Poor mental health ^a^	Ref	1.28 (0.99-1.67)	1.05 (0.80-1.36)

## Discussion

Findings from this study showed a significant association between depression and poor asthma control patterns among adults. Depression was significantly associated with poor and fair asthma control patterns among adults greater than or equal to 55 years and less than 55 years, respectively. Conversely, poor mental health status was associated with poor asthma control among adults less than 55 years.

Similar results that found an increased effect of depression on asthma control corroborated our findings [10, 29, 30, 31]. Toyama M et al. demonstrated differences in asthma control scores (ACT-J), a clinical measure of asthma control patterns between depressed and non-depressed Japanese adults. They found that the cut-off point of ACT-J in more depressive patients was lower than that in less depressive patients indicating poor asthma control patterns among depressed adults [29]. Also, Liu S et al. found a direct relationship between depression and poor asthma control [30]. Sastre J et al. reported that for patients receiving standardized asthma care in addition to routine health care visits with psychiatric specialists, asthma patients present a significant improvement in these psychological disorders and exhibit better asthma control and functional parameters [31]. Urrutia I et al., in a study, deduced a decline in asthma control and reduced quality of life among patients with asthma, anxiety, and depression, thereby indicating the need for assessment and treatment of psychological factors to improve asthma control and quality of life [10]. Some mechanisms have been proposed on how depression affects asthma control. Depression directly reduces the health-related quality of life of asthmatics [32], which in turn, affects asthma control [10]. A meta-analysis study suggested that depression and mental health disorders are associated with poor adherence to medication across a range of chronic diseases [33]; therefore, medication non-adherence could also be a pathway by which depression influences asthma control. Baiardini I et al. reported that depression was negatively correlated with acceptance of illness limitations, knowledge of the illness, and ability to identify worsening signs among asthmatics [34]. We found differences in the association between depression and mental health status by age group. Depression and poor mental health were associated with poor asthma control and fair asthma control in people under 55, while only depression was associated with poor asthma control among adults ≥55. An overall decrease in prevalence estimates for adults in the moderate asthma group could explain this observation. More so, the observed association of depression and poor asthma control was slightly higher in adults ≥55 years, although the prevalence of depression in both groups was not statistically significant. Differences in the association of depression and asthma control among both age groups could possibly be due to several factors, including a higher number and spectrum of comorbidities in older age groups compared to the younger age group [35], increased prevalence of mental disorders like depression and anxiety [36], and effects of advancing aging and risk of adverse effects of treatment in this age group [37].

Our study has several strengths. The BRFSS ACB dataset utilized in this study is an extensive and comprehensive population survey of adults with asthma in the United States. Therefore, this dataset's results reflect the current asthma prevalence in US adults. Sensitivity analysis after accounting for missing values through multiple imputations showed no difference between the listwise deleted dataset and the imputed dataset. Therefore, the sample size in this study allowed for a precise approximation of asthma control patterns using the history-elicited markers of asthma control. Although measurement invariance did not hold across the age groups, dividing the sample into two groups allowed evaluation of exposure-outcome within each group. Statistical adjustments for differences in several health and lifestyle covariates helped provide a more detailed assessment of the strength of the association. This study's limitations include using self-report measures for exposure and outcome ascertainment. Asthma control patterns were ascertained through an LCA that is data-dependent. The LCA also has limitations discussed elsewhere [26]. The cross-sectional study design does not permit assessment of the direction of the observed association, and causality cannot be inferred. The cross-sectional nature of this study also limited the scope of the secondary data analysis as some putative risk factors were not included in the survey. There is also a possibility of residual confounding due to the unavailability of confounders like disease comorbidity status. Low response rates and missing data are considered potential sources of selection bias threatening the validity of findings. Despite these limitations, this nationally representative sample provides insight into depression and poor mental health association with asthma control patterns.

## Conclusions

These findings illustrate the link between asthma, depression, and mental health status in the population and the importance of targeted prevention and integrated treatment approaches toward patients at risk or already suffering from asthma, depression, or both. A great deal of research still needs to be done to understand the role of depression and other psychopathologies on asthma control and management, as this will proffer more effective ways of caring for people with these coexisting morbidities and significantly improve patient outcomes. The association between depression and asthma control patterns has substantial public health and clinical implications, as efforts to detect, screen, and treat depression and mental health disorders would help improve asthma control, which will reduce asthma attacks, reduce asthma deaths, and reduce asthma ER visits, with the ultimate aim of meeting the Healthy People 2030 goals to improve respiratory health.
